# Loss of Wild-Type ATRX Expression in Somatic Cell Hybrids Segregates with Activation of Alternative Lengthening of Telomeres

**DOI:** 10.1371/journal.pone.0050062

**Published:** 2012-11-20

**Authors:** Kylie Bower, Christine E. Napier, Sara L. Cole, Rebecca A. Dagg, Loretta M. S. Lau, Emma L. Duncan, Elsa L. Moy, Roger R. Reddel

**Affiliations:** 1 Cancer Research Unit, Children’s Medical Research Institute, Westmead, New South Wales, Australia; 2 Children’s Cancer Research Unit, Children’s Hospital at Westmead, Westmead, New South Wales, Australia; 3 Sydney Medical School, University of Sydney, New South Wales, Australia; University of North Carolina, United States of America

## Abstract

Alternative Lengthening of Telomeres (ALT) is a non-telomerase mechanism of telomere lengthening that occurs in about 10% of cancers overall and is particularly common in astrocytic brain tumors and specific types of sarcomas. Somatic cell hybridization analyses have previously shown that normal telomerase-negative fibroblasts and telomerase-positive immortalized cell lines contain repressors of ALT activity, indicating that activation of ALT results from loss of one or more unidentified repressors. More recently, ATRX or DAXX was shown to be mutated both in tumors with telomere lengths suggestive of ALT activity and in ALT cell lines. Here, an ALT cell line was separately fused to each of four telomerase-positive cell lines, and four or five independent hybrid lines from each fusion were examined for expression of ATRX and DAXX and for telomere lengthening mechanism. The hybrid lines expressed either telomerase or ALT, with the other mechanism being repressed. DAXX was expressed normally in all parental cell lines and in all of the hybrids. ATRX was expressed normally in each of the four telomerase-positive parental cell lines and in every telomerase-positive hybrid line, and was abnormal in the ALT parental cells and in all but one of the ALT hybrids. This correlation between ALT activity and loss of ATRX expression is consistent with ATRX being a repressor of ALT.

## Introduction

Telomeres contain repetitive DNA, (TTAGGG)_n_, to which specific proteins bind and act in concert to maintain chromosome integrity [1]. In normal human somatic cells, telomeres undergo progressive shortening with cell division [2], which accounts for the observation that these cells undergo a limited number of cell division cycles before permanently ceasing proliferation and becoming senescent [3]. Immortalized human cells avoid senescence via the activity of a telomere lengthening mechanism (TLM) that counteracts the normal process of telomere attrition [4]: via either the reverse transcriptase enzyme, telomerase [5] or the alternative lengthening of telomeres (ALT) mechanism [6].

ALT occurs in a minority of cancers overall, and in more than 50% of leiomyosarcomas, osteosarcomas, astrocytic tumors grades 2 and 3, and undifferentiated pleomorphic sarcomas [7]–[9]. The types of tumors in which ALT is common are often aggressive and difficult to treat by currently available therapeutic modalities. ALT is a recombination-mediated DNA replication mechanism [10] where telomeres become lengthened via use of telomeric DNA as a copy template for replication (reviewed in [11]). An understanding of how this TLM is repressed in normal cells and activated in cancers may provide possibilities for new forms of treatment.

We have previously shown by somatic cell hybridization analyses that normal telomerase-negative fibroblasts contain one or more repressors of ALT activity [12]. Similarly, fusion of ALT and telomerase-positive cells has been shown to result in hybrid cell lines in which telomerase remained active but ALT was repressed [6], [12], [13], indicating that telomerase-positive cells contain one or more ALT repressors. Analysis of the cells in which ALT was repressed showed that there was an initial period of rapid reduction in telomere length [12], followed by a period in which the telomeres shortened at the same rate as in normal telomerase-negative cells, until finally the telomeres reached a length at which they became stably maintained by telomerase [12].

In another study, fusion of different sets of ALT and telomerase-positive cell lines produced hybrid cells in which telomerase was repressed and the features of ALT were still present [14]. The simplest explanation would be that telomerase-positive and ALT cells each contain repressors of the other TLM, and that hybrids of these cells can only continue to proliferate if they lose the repressors of either telomerase or ALT via a genetic or epigenetic event.

Recently, an important clue to the identity of ALT repressors was provided by the observation that pancreatic neuroendocrine tumors commonly contain inactivating mutations in either ATRX or DAXX [15]. Subsequently, all of the tumors containing these mutations were characterized as being ALT-positive by the presence of ultra-bright telomere signals, whereas all of the tumors with normal nuclear ATRX and DAXX were ALT-negative [16]. ALT-positive tumors of the central nervous system were also shown to have ATRX mutations or to lack ATRX staining [16], [17]. Furthermore, ATRX expression was found to be impaired in 19 of 22 ALT cell lines [18]. Loss of ATRX/DAXX function has therefore been proposed to be involved in activating ALT [16], [18].

To test this hypothesis, we analyzed the TLM in hybrid cell lines formed by fusing one ALT cell line with four different telomerase-positive cell lines and determined whether this correlated with normal expression of DAXX and ATRX. In the great majority of the hybrid clones, one or other TLM was repressed, and repression of ALT correlated with wild-type ATRX expression. These data are consistent with ATRX being an ALT repressor.

## Materials and Methods

### Cell Lines

The universal hybridizer ALT cell line, GM847DM (hereinafter referred to as GM847), derived from simian virus 40 (SV40)-immortalized Lesch-Nyhan syndrome male human fibroblasts, were provided by Dr. Olivia Pereira-Smith [19]. Telomerase-positive cell lines A549 (male), J82 (male) and TE-85 (female) were obtained from ATCC (Manassas, VA) and MeT-5A cells (male) were obtained from Dr. Curtis Harris (National Cancer Institute, Bethesda, MD; [20]). GM02063 mortal cells, from which GM847 cells were derived, were obtained from Coriell Institute for Medical Research. All parental cell lines and hybrid cell lines were maintained in Dulbecco’s modified Eagles medium (DMEM; Life Technologies) supplemented with 10% fetal bovine serum (FBS; Sigma) in a 37°C humidified 5% CO_2_ incubator.

Generation of the MeT-5A/GM847 hybrids was described previously [21], and A549/GM847, J82/GM847, and TE-85/GM847 hybrid cell lines were generated by the same methods. Briefly, equal numbers of GM847 and telomerase-positive cells were mixed and then fused by adding polyethylene glycol. Fused cells were selected in HAT-supplemented DMEM containing 10% FBS and 10^−6^M ouabain.

### Telomere Repeat Amplification Protocol (TRAP)

The TRAP assay was performed essentially as described previously [22], [23]. Briefly, lysates were prepared from equal cell numbers using CHAPS lysis buffer. Telomerase activity was indicated by the presence of a 6 base pair ladder when the PCR amplification products were separated by 10% non-denaturing, polyacrylamide gel electrophoresis. Gels were stained with SYBR Gold (Life Technologies) and PCR products visualized using a Typhoon imager (GE Healthcare).

### Terminal Restriction Fragment (TRF) Analysis

TRF analysis was performed as described previously [12]. Genomic DNA was digested with the restriction enzymes *Hinf*I and *Rsa*I (New England Biolabs) and 1 µg of digested DNA subjected to pulsed field gel electrophoresis in 0.5x TBE buffer. The gels were dried, denatured, hybridized to a telomeric-specific oligonucleotide probe, and exposed overnight to a PhosphorScreen (GE Healthcare). The PhosphorScreen was then scanned using a Typhoon imager.

### C-circle Assay

The C-circle assay was performed using 30 ng of genomic DNA and the signal was detected using a γ-^32^P-labeled-(CCCTAA)_3_ oligonucleotide probe as previously described [24]. Results from two separate experiments were quantified using ImageQuant software, with array analysis and edge subtraction used for background correction.

### Antibodies

The following primary antibodies were used for indirect immunofluorescence and/or immunoblotting: ATRX (Sigma), DAXX (Sigma), γ-tubulin (Sigma), TRF2 (Millipore), and PML (Santa Cruz). Secondary antibodies for immunofluorescence were obtained from Life Technologies: Alexa Fluor 488 donkey anti-mouse IgG, Alexa Fluor 488 goat anti-rabbit IgG, Alexa Fluor 594 donkey anti-goat IgG, and Alexa Fluor 647 donkey anti-rabbit IgG. The secondary antibody goat anti-mouse HRP used for immunoblotting was obtained from Dako.

### Immunofluorescence Analysis

For immunodetection of ATRX and DAXX, cells fixed with 4% paraformaldehyde were permeabilized twice with 0.1% Triton X-100. ATRX and DAXX antibodies were used at a dilution of 1∶200 in 3% BSA/PBS. Reacting antibodies were detected using Alexa Fluor 488 conjugated secondary antibodies at a dilution of 1∶1,000 and cells were counterstained with DAPI (Sigma). Images were captured on an Axio-Imager M1 microscope (Carl Zeiss) using a 40x lens and analysed using AxioVision software (Carl Zeiss).

### Immunoblotting

Immunoblotting was performed on whole cell extracts using standard techniques. Briefly, 30 µg of protein lysate was separated on a 3–8% NuPAGE Tris-Acetate polyacrylamide SDS gel (Life Technologies), transferred to PVDF membrane (Millipore), and probed with ATRX, DAXX, and γ-tubulin antibodies. Detection was performed using SuperSignal West Pico chemiluminescent substrate (Pierce) and analyzed on an ImageQuant LAS 4000 Biomolecular Imager (Fujifilm).

### DNA Sequencing of ATRX, DAXX, and H3.3 and Single Nucleotide Polymorphism (SNP) Analysis of ATRX

All ATRX and DAXX exons were amplified using primers and PCR conditions as described [15]. H3.3 was sequenced using the following primer sets: exon 1: forward 5′ GTGATCGTGGCAGGAAAAGT and reverse 5′ AGCAAAAAGTTTTCCTGTTATCCA; exon 2: forward 5′ TTTCTTTGAAGCTGCCCACT and reverse 5′ AGCACATGCAACAATTTGGA, and exon 3: forward 5′ GCATCTTGCCCAGTCATTTT and reverse 5′ TGGAAAAACTGCCAATACCTG. PCR products were purified with the QIAquick PCR Purification Kit (Qiagen) and subjected to Sanger sequencing (Australian Genome Research Facility). The parental origin of the ATRX alleles in the hybrids was identified using SNP rs3088074 located on exon 9.

### Immunodetection of ALT-associated PML Bodies (APBs)

For immunodetection of APBs, cells fixed with 4% paraformaldehyde were permeabilized with methanol. Cells were then incubated concurrently with primary antibodies TRF2, PML, and ATRX at dilutions of 1∶300, 1∶100, and 1∶500, respectively, followed by secondary antibodies to detect the protein in parentheses: Alexa Fluor 488 (TRF2) and Alexa Fluor 594 (PML) at a dilution of 1∶1,000 and Alexa Fluor 647 (ATRX) at a dilution of 1∶500. Images were captured on an Axio-Imager M1 microscope using AxioVision 4.8.2 software (Carl Zeiss) and appropriate filter sets. Nuclei containing colocalized TRF2 and PML staining were considered positive for the presence of APBs.

## Results

### Growth of Hybrid Cell Lines

The ALT cell line, GM847, was fused with four different telomerase-positive cell lines, and clonally derived hybrid cell lines were obtained by marker selection. The hybridization efficiency and the frequency with which immortal hybrid lines arose for each set of hybrid lines is indicated in Table S1. Hybrid lines are named with the first letter (X) of the telomerase-positive parent cell line name and a letter (Y) identifying the clone in the format X-Y.

**Figure 1:**
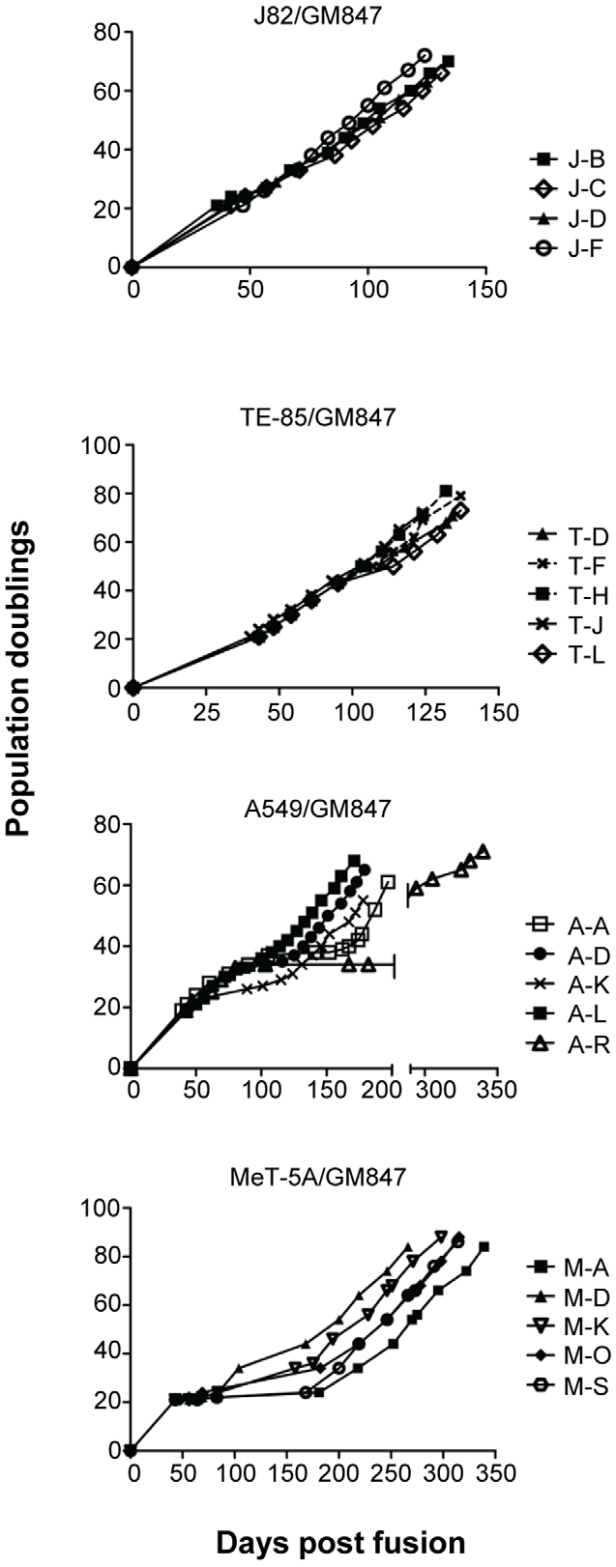
Growth curves of hybrid cell lines. GM847 cells were fused with J82, TE-85 or A549 telomerase-positive cell lines using polyethylene glycol. Proliferation of each hybrid clone was plotted as a function of population doubling and the number of days following fusion. Growth of MeT-5A/GM847 hybrid lines has been described previously [21].

We previously showed that fusing MeT-5A with GM847 resulted in 18 of 18 hybrid lines ceasing proliferation after 20–45 population doublings (PD), and that 13 eventually recommenced proliferation after 50–100 days (Figure 1; Table S1) [21]. Five of the MeT-5A/GM847 hybrid lines were analyzed further here. Four or five hybrid lines generated from the fusions between GM847 and TE-85, J82 or A549 cells were passaged continuously for 150–350 days to determine their proliferative capacity. Like the MeT-5A/GM847 hybrids, some of these hybrid lines proliferated for 25–50 PDs before entering a period of senescence-like growth arrest or crisis (Figure 1; Table 1; Table S1). All of the hybrid lines studied here eventually recommenced proliferation, presumably due to the appearance of an immortal segregant within the population. Some of the hybrid lines had little or no evidence of growth arrest, indicating either that they did not complement for the mortal phenotype [19], or that an immortal segregant emerged rapidly following fusion. The subsequent analyses were performed in cell lines when they were presumed to be immortal, i.e., at PD levels that were greater than the highest PD level at which any hybrid line emerged from growth arrest.

**Table 1:** Correlation between ALT activity and lack of normal ATRX expression.

Cell Line		Growth Arrest (days)^a^	TRAPAssay^b^	TRF^c^	CCAssay^d^	TLM^e^	ATRXprotein^f^	ATRXSNP^g^	ATRXExon 1^i^
GM847		N/A	–	+	+	ALT	–	G	–
J82		N/A	+	–	–	TEL	+	C	+
TE-85		N/A	+	–	–	TEL	+	G	+
A549		N/A	+	–	–	TEL	+	C	+
MeT-5A		N/A	+	–	–	TEL	+	C	+
J82/GM847	J-B	0	+	–	–	TEL	+	C/G	+
	J-C	0	+	–	–	TEL	+	C/G	+
	J-D	0	+	–	–	TEL	+	C/G	+
	J-F	0	+	–	–	TEL	+	C/G	+
TE-85/GM847	T-D	0	+	–	–	TEL	+	nd	+
	T-F	0	+	–	–	TEL	+	nd	+
	T-H	0	+	–	–	TEL	+	nd	+
	T-J	0	+	–	–	TEL	+	nd	+
	T-L	0	+	–	–	TEL	+	nd	+
A549/GM847	A-A	57	–	+	+	ALT	+ (low)	C/G^h^	+
	A-D	27	+ (low)	–	–	TEL	+	C/G	+
	A-K	59	–	+	+	ALT	+ (low)	C/G^h^	+
	A-L	0	–	+	+	ALT	–	G	–
	A-R	102	–	+	+	ALT	+	C/G	+
MeT-5A/GM847	M-A	138	–	+	+	ALT	–	G	–
	M-D	41	–	+	+	ALT	–	G	–
	M-K	132	–	+	+	ALT	–	G	–
	M-O	139	+	–	–	TEL	+	C	+
	M-S	125	+	–	–	TEL	+	C	+

a 6/7 ALT hybrids underwent a period of growth arrest compared to 3/12 TEL hybrids (p = 0.0198, Fisher’s exact test);

b TRAP assay: “+” indicates detectable telomerase activity (Figure 2);

c TRF: “+” indicates a terminal restriction fragment length pattern characteristic of ALT (Figure 3);

d CC assay: “+” indicates C-circle levels above the cut-off level for ALT activity (Figure 4);

e TLM: telomere lengthening mechanism deduced from TRAP, TRF and CC assay data; ALT and TEL indicate ALT-positive and telomerase-positive, respectively;

f ATRX protein: “+:” indicates normal pattern of immunofluorescence (Figure 5) and normal levels on Western (Figure 6);

g ATRX SNP: a single nucleotide polymorphism (rs3088074, c.3017C>G, p.Q929E) for which each of the parental lines is homozygous identified the parental origin(s) of the ATRX alleles in all hybrids except the TE-85/GM847 lines for which the SNP was not determined (nd), because TE-85 and GM847 both have G at this location;

h The magnitude of the C peak was significantly smaller than that of the G peak;

i ATRX exon 1: “+” indicates the presence of wild-type ATRX exon 1 as determined by PCR (Figure 7).

### Telomerase Activity

Telomerase activity (Figure 2; Table 1) was detected using the TRAP assay [22] and was detected in every hybrid cell line in the sets derived from fusion of GM847 with J82 or TE-85. The A549/GM847 and MeT-5A/GM847 hybrids, however, were not all telomerase-positive. Telomerase activity was clearly detected, although at a relatively low level, in only one of the five A549/GM847 lines (A-D), while the remaining hybrids were negative for telomerase activity. Two of the five MeT-5A/GM847 hybrid lines (M-O and M-S) were positive for telomerase activity, while the other three showed no detectable activity.

**Figure 2:**
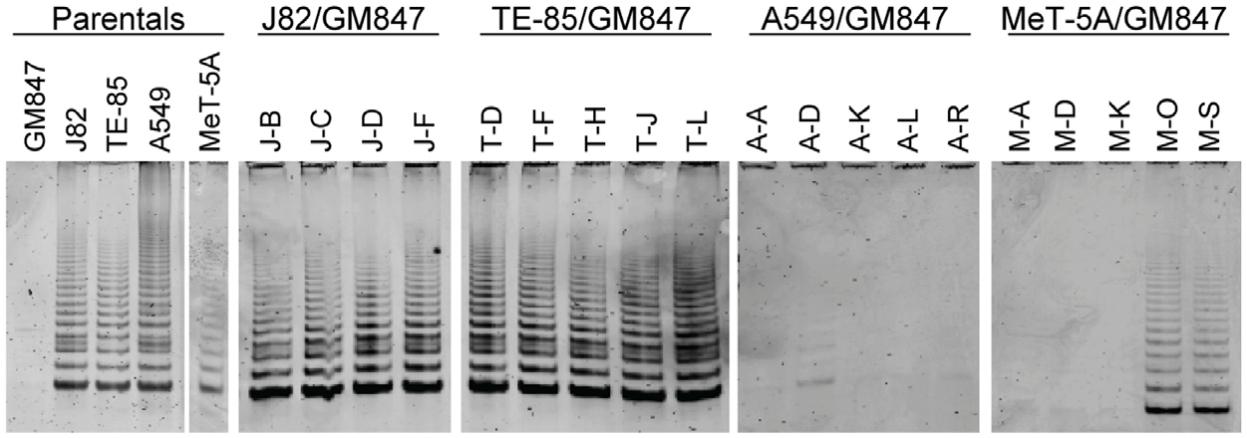
Characterization of the TLM of the hybrid clones by telomerase activity assay. Telomerase activity of parental and hybrid cell lines using TRAP analysis. Presence of a six base pair ladder indicates the expression of telomerase.

### Telomere Length Analyses

**Figure 3:**
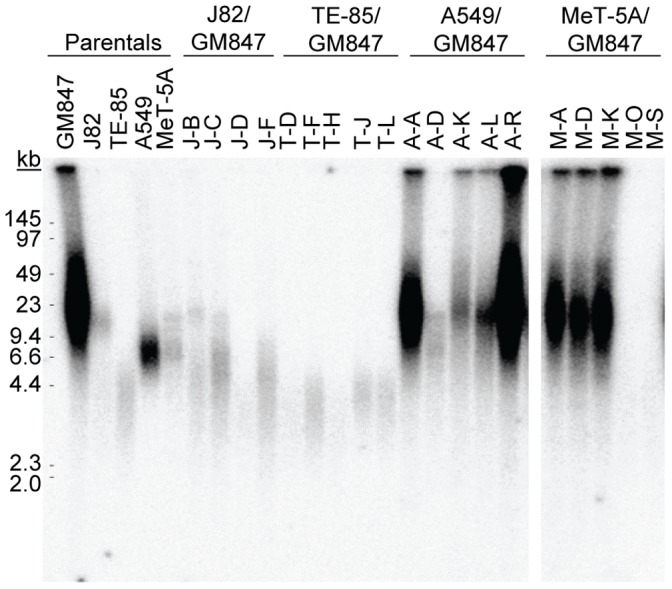
Assessment of telomere length using TRF analysis. Long, heterogeneous telomere lengths are indicative of ALT, while shorter, more homogeneous telomere lengths indicate the cells lines are ALT-negative. A single gel is shown and the space indicates where irrelevant lanes have been removed.

Most human ALT cell lines examined to date have a characteristic pattern of telomere lengths, ranging from very short to extremely long [6], [25]. TRF analyses for each of the hybrids and the parental cell lines from which they were derived are shown in Figure 3 and summarized in Table 1. The hybrids formed between GM847 and J82 or TE-85 cells all had telomere lengths similar to, or less than, their telomerase-positive parental counterpart, consistent with all of these lines being telomerase positive. All of the A549/GM847 hybrids, except line A-D, which was telomerase positive, contained long, heterogeneous telomeres very similar to those of the ALT parental cell line, GM847. Similarly, the three telomerase-negative MeT-5A/GM847 hybrid lines had long, heterogeneous telomeres, whereas the two telomerase-positive MeT-5A/GM847 hybrids had telomere lengths characteristic of telomerase-positive lines.

In all of the hybrid lines examined, therefore, there was a clear-cut correlation between telomerase status and telomere length pattern: the hybrids either had telomerase activity and a TRF pattern consistent with telomere maintenance via telomerase, or they were telomerase-negative and had the long, extremely heterogeneous TRF pattern characteristic of ALT. No evidence was found for the persistence of both TLMs in any hybrid line. The hybrid cell lines that were ALT-positive were significantly more likely to have undergone a period of growth arrest (Table 1).

### C-circle Assays

**Figure 4:**
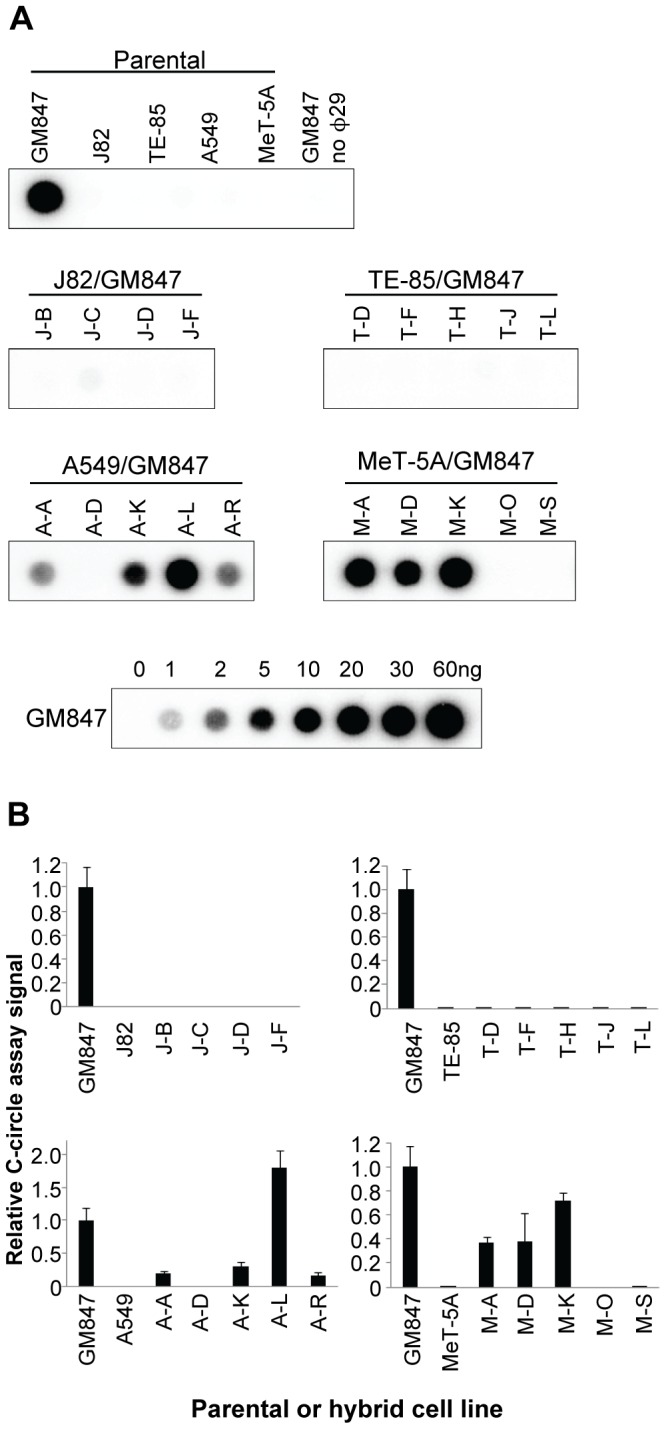
Detection of ALT activity by C-circle assay. A. Dot blot of C-circle assay products for each parental and hybrid line. Serial dilutions of genomic DNA from GM847 cells indicate linearity of the assay within this range. The presence of C-circles indicates the cell line utilizes the ALT mechanism. B. Quantitation of C-circle assay levels by densitometry. The results from two separate experiments are presented relative to the signal obtained by GM847 cells. Bars, average; error bars, range.

The parental and hybrid cell lines were all analyzed for C-circles (Figure 4), which are partially single-stranded telomeric circles in which the C-strand is intact and which appear to be specific for ALT [24]. The C-circle results correlated with the TRF and the telomerase results in all parental and hybrid cell lines, without any exceptions. Every line that had a TRF pattern indicative of ALT was positive for C-circles. Furthermore, all hybrids that were telomerase-positive or -negative were C-circle-negative or -positive, respectively. Three telomerase-negative hybrid A549/GM847 lines (A-A, A-K, and A-R) had lower C-circle levels than the other ALT-positive hybrid lines, but were nevertheless clearly ALT-positive. It was therefore possible to unambiguously categorize each cell line as either ALT-positive or telomerase-positive (Table 1).

### Correlation between ALT and Loss of Nuclear ATRX foci

**Figure 5:**
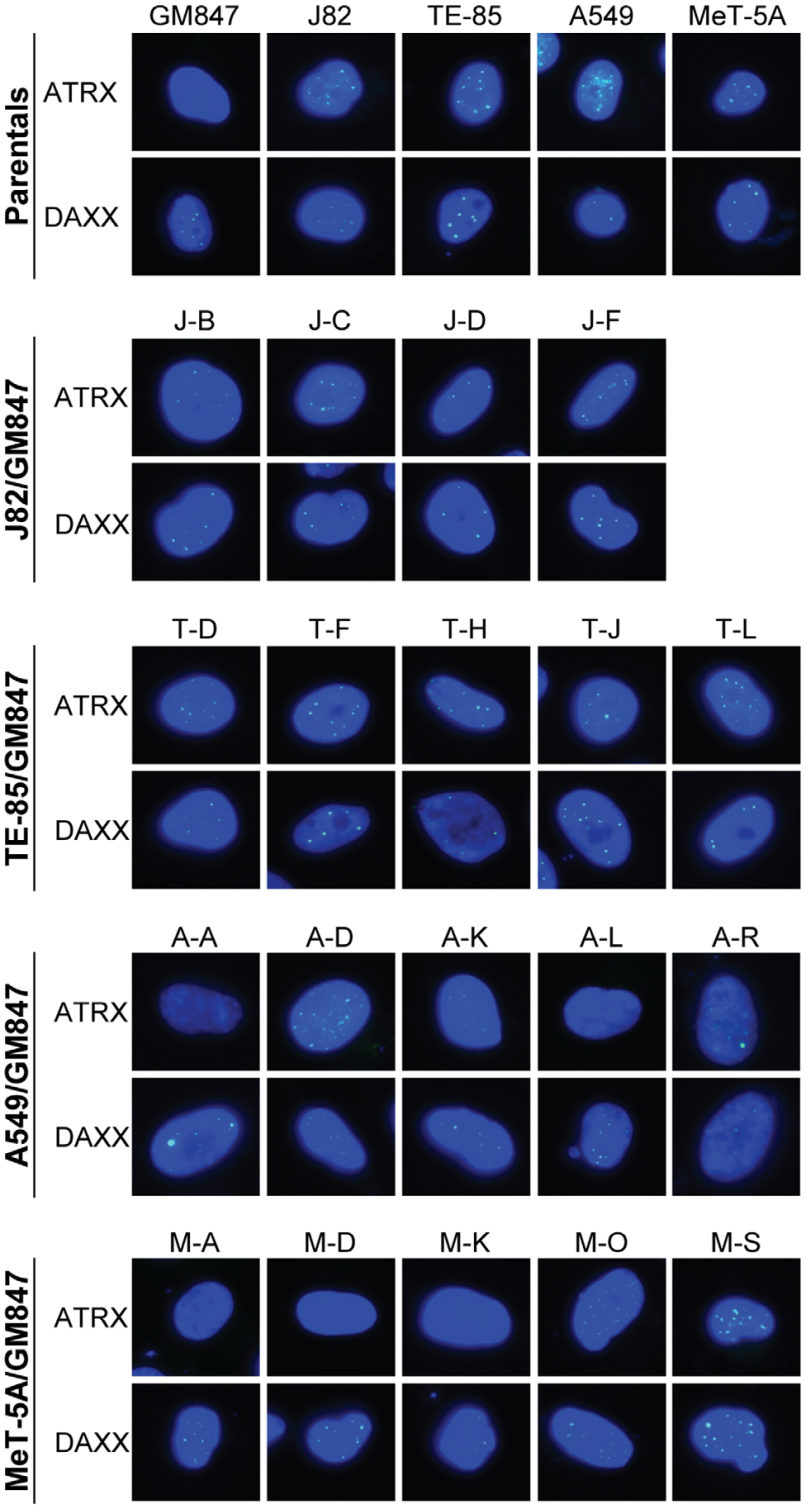
Indirect immunofluorescent staining of ATRX and DAXX in parental and hybrid cell lines. Representative images of nuclei stained with ATRX or DAXX antibodies (green) and DAPI (blue) are shown for each cell line, with two exceptions: for hybrid lines A-A and A-K only 20% and 8%, respectively, had ATRX foci.

ATRX and DAXX can be visualized using immunofluorescence as foci that colocalize with PML bodies within the nucleus [26]. Representative nuclei are shown in Figure 5 and the data are summarized in Table 1. DAXX foci were detected in every parent and hybrid cell line although the intensity of the signal was variable. ATRX foci were present in telomerase-positive parental and hybrid lines, and absent from the majority of ALT-positive hybrid cell lines. Although the correlation between ALT activity and absence of ATRX foci was excellent overall, the situation was not as clear-cut for three of the A549/GM847 hybrids. Lines A-A and A-K had lost ATRX nuclear staining in the majority (80 and 92%, respectively) of cells. In contrast, line A-R had ATRX foci in the majority (>95%) of nuclei in the population.

### ATRX Protein Levels

**Figure 6:**
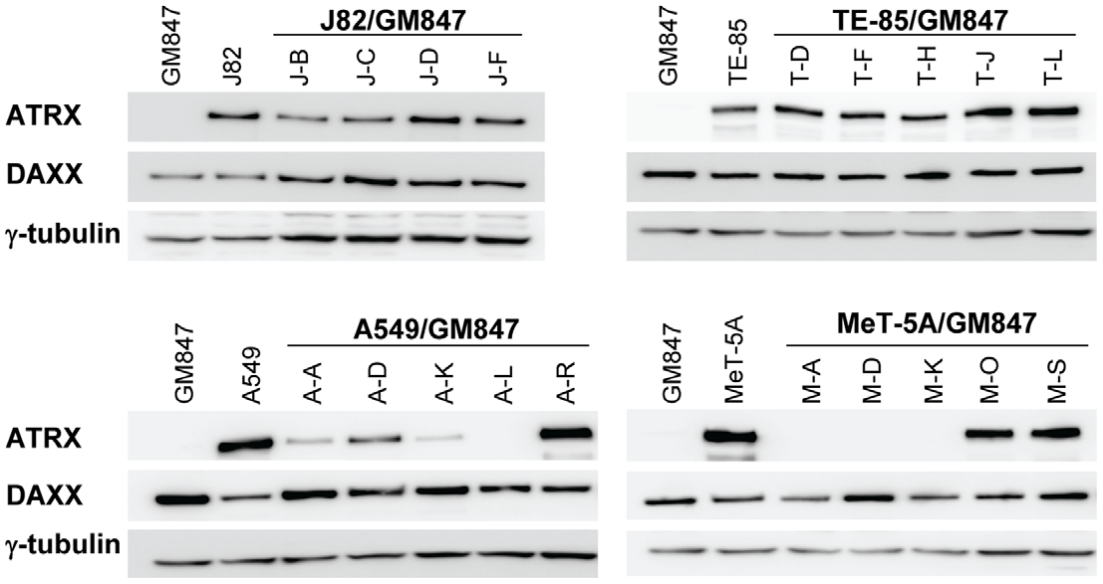
ATRX and DAXX protein expression in parental and hybrid cell lines. Immunoblots for each cell line were probed with the antibody indicated to the left of each blot. Staining with γ-tubulin antibody was used as a loading control.

ATRX immunoblotting results (Figure 6 and Table 1) correlated with immunostaining: in every case where nuclear ATRX staining was lost, ATRX protein was not detected in whole cell extracts. The two ALT-positive hybrid A549/GM847 lines, A-A and A-K, that had minor subpopulations with ATRX foci had very low ATRX levels. A third, A-R, had high levels of ATRX protein, consistent with the observation that the great majority of these cells had ATRX foci.

### Detection of APBs and ATRX Foci

Another hallmark of ALT activity is the presence of APBs, which are large aggregates of telomeric DNA and telomere binding proteins within PML nuclear domains, in a subset of cells within the population [27]. We visualized APBs and ATRX foci simultaneously in the A549/GM847 A-K line that contained a mixture of cells with and without ATRX foci. Of >500 cells that were examined, 34% contained APBs and 8% contained ATRX foci (data not shown). If the distribution of APBs and ATRX were random, 8% of the cells that contained APBs would also be expected to show ATRX foci; however, no colocalization of ATRX foci and APBs was detected.

### ATRX Sequence and Correlation between ATRX Expression and Origin of ATRX Alleles

In order to determine the parental origin of the ATRX allele in the hybrid lines, we identified an ATRX sequence polymorphism (SNP). We found a SNP for which each of the parental cell lines was homozygous, and for which the GM847 allele (“G” SNP) differed from the allele in three of the four telomerase-positive parental lines (“C” SNP), and were therefore able to deduce the parental origin of the ATRX gene in three of the four sets of hybrid lines (Table 1). We found that all hybrid lines which lacked ATRX expression were homozygous for the G SNP, meaning that the ATRX allele from the telomerase-positive parental cell line was lost from the hybrid, whereas the ATRX allele from the ALT-positive parent was retained.

**Figure 7:**
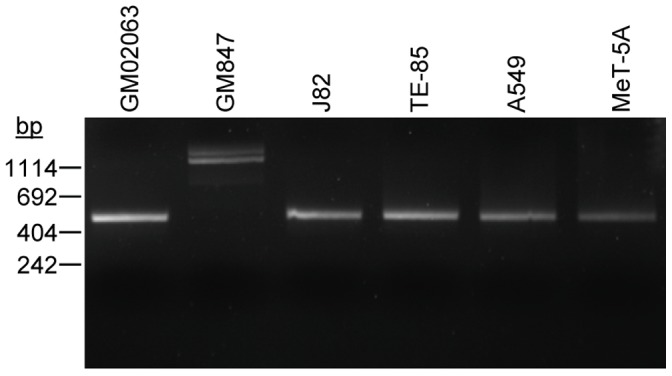
Agarose gel electrophoresis of ATRX exon 1 PCR products. ATRX exon 1 of each of the indicated samples was amplified by PCR as described in the Materials and Methods. The PCR products (expected size 418bp) were subjected by agarose gel electrophoresis and verified by Sanger sequencing. GM02063 is the parent cell strain from which GM847 cells were derived by SV40-induced immortalization.

We then sequenced all exons of ATRX in each parental cell line, as well as the cell strain (GM02063) from which GM847 cells were derived and found that GM847 cells lack exon 1 of ATRX (Figure 7). The amplified band from GM847 cells was sequenced and found to be unrelated to ATRX. Exon 1 of ATRX was also missing from each of the ALT-positive hybrid lines that were homozygous for the G SNP, confirming that these hybrids only contained an ATRX allele from the GM847 parent cell line (Table 1). Conversely, all hybrid clones that expressed ATRX were either homozygous for the C SNP or heterozygous (C/G), indicating that the presence of one or more C alleles from the telomerase-positive parent was sufficient to result in a wild-type ATRX expression pattern, and to suppress ALT, regardless of whether one or more ALT-associated alleles were present. Moreover, in two of the eight hybrid lines that were heterozygous for the SNP (A-A and A-K), the chromatographs showed that the magnitude of the C peak was significantly smaller than that of the G peak, and this correlated with the very low level of ATRX expression in these two lines. We examined whether inactivating mutations in ATRX, DAXX or H3.3 were responsible for the high level of ATRX expression and normal ATRX foci in A-R by sequencing all exons in each of these three genes and found that hybrid line A-R did not harbor any mutations in ATRX, DAXX or H3.3.

## Discussion

**Figure 8:**
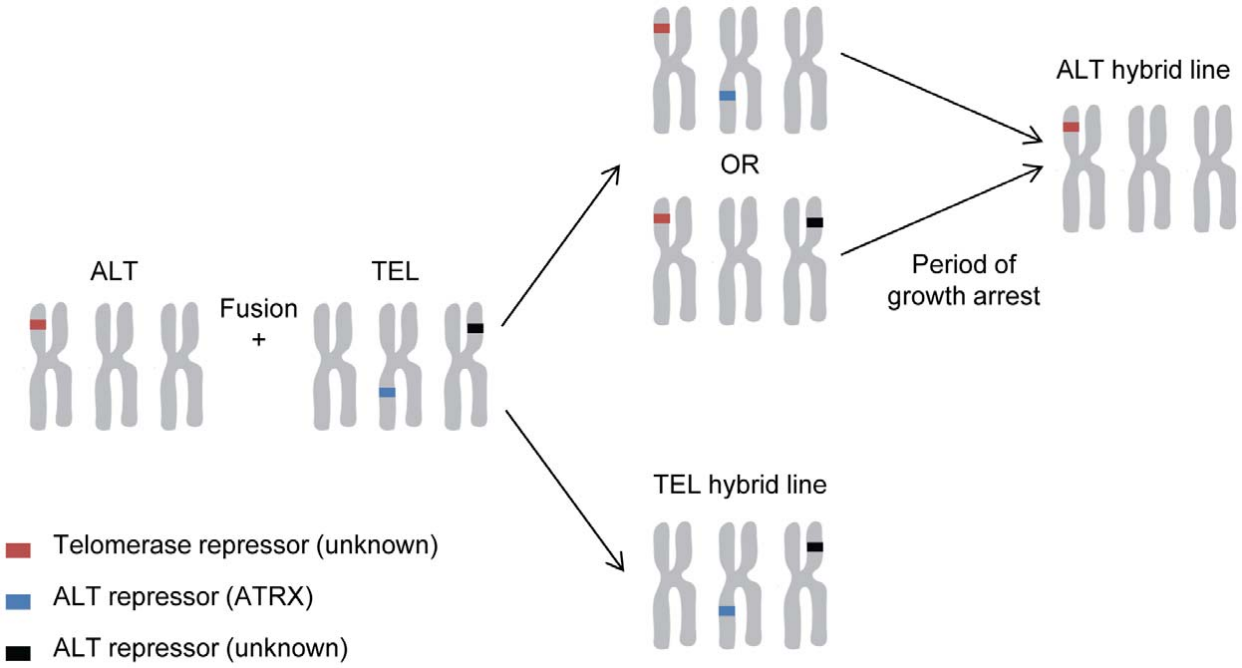
Schematic representation of TLM activation in somatic cell hybrids. We propose that ALT and telomerase-positive cell lines contain repressors of the other TLM and that fusion of an ALT and a telomerase-positive cell line therefore results in suppression of both TLMs. In this model, activation of telomerase results from loss of expression of a putative telomerase repressor, by a genetic or epigenetic mechanism. The earliest PD level at which growth arrest occurred in any hybrid was 25 PD, but in the chromosomally unstable environment of a somatic cell hybrid this is a sufficient number of cell divisions for the population to be overgrown by cells in which one or two repressors have been lost, so that no growth arrest is observed. Activation of ALT is associated with loss of ATRX expression, and the association between ALT and growth arrest suggests that activation of ALT requires more events than activation of telomerase in the hybrids.

We used somatic cell hybridization analysis to determine whether repression of ALT is associated with normal expression of ATRX/DAXX, because it was this experimental approach that had previously shown normal cells and telomerase-positive cell lines contain repressors of ALT [6], [12], [13], [23]. It has also been shown that normal cells and ALT cell lines contain repressors of telomerase [14], [28]. Following cell fusion, chromosomes are lost from the resulting hybrid such that the DNA content of the hybrid is less than the sum of the parents [29], so the TLM used by the hybrid cells may be determined by the relative copy number of genes that repress either mechanism, or other factors that influence the probability that expression of the repressor(s) of one or other TLM will be lost from the hybrid. The existing data support the conclusion that normal somatic cells contain repressors of both TLMs, and that ALT-positive and telomerase-positive immortalized cells lose repressors of ALT and telomerase, respectively, but retain the normal repressors of the other mechanism. Our results support this conclusion since we found that in hybrids of telomerase-positive and ALT-positive cell lines, one or other mechanism was repressed. The simplest explanation for this observation would be that following formation of hybrid cells that contain repressors of both ALT and telomerase, the only cells that survive are those that have lost repression of either ALT or telomerase, and once this has occurred there is no further selection pressure to lose repression of the other TLM (Figure 8).

The evidence that normal mortal cells contain repressors of ALT and telomerase indicates that it is very unlikely that ALT and telomerase are each other’s repressors. This has been verified experimentally by expressing telomerase exogenously in ALT cells and showing that the two mechanisms can co-exist within the same cell [23], [30]–[32].

Here we addressed the question whether ATRX/DAXX represses ALT. DAXX expression was normal in each of the 24 cell lines. We found a clear-cut correlation between the ATRX status and TLM in 21/24 cell lines studied (5/5 parental and 16/19 hybrid lines), and the correlation appeared to hold also in two of the remaining three lines which appeared to contain mixed populations. ATRX was normal in each of the 16 telomerase-positive lines (four parental and 12 hybrid lines) and abnormal in the ALT-positive GM847 parent and four ALT hybrid lines. A further two hybrid A549/GM847 lines, A-A and A-K, were telomerase-negative/ALT-positive by all criteria, but had low levels of ATRX protein and a small proportion of cells had ATRX foci (20% and 8%, respectively). These two lines thus appear to contain distinct subpopulations, with the majority being ATRX-negative/ALT-positive. Consistent with this, when we examined APBs and ATRX foci in the A-K line, we did not observe any cell that contained both. Moreover, SNP analyses of the A-A and A-K lines indicated that the ATRX allele from the telomerase-positive A549 parent line was present at very low abundance. These lines were TRAP-negative, and given that the TRAP assay is usually sensitive enough to detect minor populations of telomerase-positive cells it seems likely that the subpopulations of ALT-negative cells are also telomerase-negative. Presumably these cells have telomeres that are sufficiently long to support continued proliferation in the absence of a TLM; a previous study using a different ALT cell line demonstrated that some cells were able to continue proliferating for 60 to 80 PDs after ALT activity was suppressed [33]. Subcloning of hybrid lines A-A and A-K would aid in characterizing the distinct ATRX-negative and ATRX-positive subpopulations.

The data overall are therefore consistent with the hypothesis that ATRX is a repressor of ALT [16], [18], which involves recombination-mediated replication of telomeric DNA [10]. A potential mechanism for ATRX repression of ALT may be alteration of telomeric chromatin in such a way that telomeric recombination is repressed. ATRX belongs to the SWI/SNF family of ATP-dependent chromatin remodeling proteins [34], [35] and together with DAXX is thought to be involved in modification of chromatin structure at heterochromatic regions including tandem repetitive DNA such as rDNA repeats and telomeres [26], [36]–[39]. Loss of ATRX function can lead to dysregulation of genes associated with tandem repeat DNA [38]. ATRX interacts with HP1α, a heterochromatin protein involved in telomere stability [40] and APB formation [41]. ATRX/DAXX has been reported to be involved in recruitment of histone H3.3 to telomeres, and loss of ATRX function results in a DNA damage response and de-repression of telomeric transcription [26], [38], [42]–[44]. The observation that ATRX/DAXX mediates recruitment of H3.3 to telomeres is consistent with the finding that mutations in H3.3 may also be associated with ALT in pediatric glioblastomas [17], [45], which suggests that ATRX, DAXX, and H3.3 act in the same pathway to repress ALT.

Although our data are consistent with ATRX being involved in repression of ALT, the low frequency with which human cells become immortalized [46] suggests that a single genetic event resulting in loss of ATRX must be insufficient for immortalization via activation of the ALT pathway and that other molecular alterations are required. This conclusion is supported by the recent finding that repression of ATRX alone in BJ fibroblasts does not result in immortalization [18]. The association between growth arrest and ALT in hybrids observed here suggests that activation of ALT requires more genetic or epigenetic events than activation of telomerase in hybrids (Figure 8). There have been no reports of an increased incidence of cancer, ALT or otherwise, in individuals with ATRX mutations [47], [48], but this may be because of the small number of such cases.

The only cell line that was an exception to the correlation between ALT and loss of ATRX expression was the ALT-positive A549/GM847 A-R line, which had a high level of ATRX and had ATRX foci in the great majority of cells. This hybrid line retained alleles from both parent lines, as indicated by ATRX SNP analysis and the presence of exon 1 of ATRX. We looked for a loss-of-function mutation in the ATRX allele from the telomerase-positive parent, but sequence analysis indicated that ATRX remained wild-type in this hybrid line, as did DAXX and H3.3. ALT cell lines with wild-type ATRX, DAXX and H3.3 have also been observed previously [18], and it will be of great interest to determine how these cells activate ALT.

## Supporting Information

Table S1 Summary of hybridization efficiency and outcomes of isolated colonies.
(DOCX)
