# Supplementary material for: Loss of Wild-Type ATRX Expression in Somatic Cell Hybrids Segregates with Activation of Alternative Lengthening of Telomeres
Source: PLoS One. 2012 Nov 20;7(11):e50062. doi: 10.1371/journal.pone.0050062 (PMC3502299; doi:10.1371/journal.pone.0050062)
Supplement: Table S1 — Summary of hybridization efficiency and outcomes of isolated colonies. (DOCX) [file pone.0050062.s001.docx]

**Table S1.** **Summary of hybridization efficiency and outcomes of isolated colonies.**

|  |  |  | Immortalized hybrid lines | |  |
| --- | --- | --- | --- | --- | --- |
| Hybrid | Hybridization efficiency^a^ | Hybrid colonies isolated | Hybrid lines without a senescence-like phase^b^ | Hybrid lines with a senescence-like phase^b^ | Non-immortalized hybrids |
| J82/GM847 | 1 in 2x10^3^ | 11 | 9 | 1 | 1 |
| TE-85/GM847 | 1 in 2x10^3^ | 11 | 9 | 1 | 1 |
| A549/GM847 | 1 in 9x10^3^ | 12 | 2 | 8 | 2 |
| MeT-5A/GM847 | 1 in 9x10^3^ | 18 | 0 | 13 | 5 |

^a^Hybridization efficiency was calculated as the number of isolated colonies per total cells seeded and subjected to selection;

^b^Senescence-like phase was defined as failure to undergo one PD within 21 days.
